# The Impact of Decreased GSK3β and S6K1 Expression in TNBC Patients

**DOI:** 10.3390/life15121917

**Published:** 2025-12-15

**Authors:** Tijana Tomić, Mirjana Prvanović, Jovan Jevtić, Blagoje Murganić, Nejla Ademović, Milica Nedeljković, Irena Jovanić, Nikola Tanić, Nasta Tanić

**Affiliations:** 1 Department of Radiobiology and Molecular Genetics, Institute of Nuclear Sciences “Vinča”, National Institute of Republic of Serbia, University of Belgrade, 11000 Belgrade, Serbia; tijana.tomic@vin.bg.ac.rs (T.T.); blagoje@vin.bg.ac.rs (B.M.); 2 Institute of Pathology, Faculty of Medicine, University of Belgrade, 11000 Belgrade, Serbia; mirjanaprvanovic@gmail.com (M.P.); jovan.jevtic@med.bg.ac.rs (J.J.); 3 Department of Neurobiology, Institute for Biological Research “Siniša Stanković”, National Institute of Republic of Serbia, University of Belgrade, 11000 Belgrade, Serbia; nejla.ademovic@ibiss.bg.ac.rs (N.A.); nikolata@ibiss.bg.ac.rs (N.T.); 4 Department of Experimental Oncology, Institute of Oncology and Radiology of Serbia, 11000 Belgrade, Serbia; mnedel30@tutanota.com; 5 Department of Pathology, Institute of Oncology and Radiology of Serbia, 11000 Belgrade, Serbia; jovanicirena@gmail.com; 6 Department of Natural Sciences and Mathematics, Field of Biology, State University of Novi Pazar, 36300 Novi Pazar, Serbia

**Keywords:** TNBC, GSK3β, S6K1, mRNA expression, IHC

## Abstract

Breast cancer is the most frequent and lethal type of cancer that affects women worldwide. Triple-negative breast cancer (TNBC) is the most aggressive type of breast cancer, having high rate of recurrence, metastasis, and mortality, with very limited options for treatment, and a tendency to develop resistance to conventional therapy. These circumstances mean that it is necessary to develop effective therapies for TNBC patients which would circumvent resistance mechanisms. The PAM and Wnt signaling pathways are among those responsible for therapy resistance in TNBC, as they also have major roles in different cellular processes such as metabolism, proliferation, metastasis, stemness, and survival. We analysed the expression of GSK3β and S6K1 as interacting components of the two pathways in order to examine the relation between them and determine whether they could be used as predictive markers in TNBC. The expression of mRNA was examined with real-time PCR and protein expression with immunohistochemistry. Our results showed that protein expression is in line with mRNA expression. We found a positive correlation between the mRNA expressions of GSK3β and S6K1, showing their coordinated transcription. We also showed that their simultaneous low expression is unfavorable for TNBC patients and could possibly be used as a predictive marker.

## 1. Introduction

Breast cancer is the most common type of malignancy and the leading cancer-related cause of death in women worldwide [1]. While breast cancer is a highly heterogenous disease, its main subtypes are defined by the presence of estrogen receptors (ERs), progesterone receptors (PRs), and human epidermal growth factor 2 receptors (HER-2). Accordingly, triple-negative breast cancer (TNBC) is characterized by the absence of ERs, PRs, and HER-2 and it accounts for 15–20% of all breast cancer cases [2]. In comparison to other types of breast cancer, TNBC is more aggressive, and has higher recurrence, metastasis, and mortality rates [3]. The absence of ERs, PRs, and HER-2 receptors makes TNBC the most challenging type of breast cancer in terms of treatment. Numerous clinical trials for targeted therapy of TNBC have not yet shown expected results, which is mainly a consequence of the high heterogeneity of TNBC [4]. The only available therapy for most TNBC patients is conventional chemotherapy, mainly anthracycline- and taxane-based chemotherapy, to which the cancer readily develops resistance. Several mechanisms that enable cancer to develop resistance have been discovered so far [5]. Aberrant signaling in both the PI3K/AKT/mTOR (PAM) and Wnt/β-catenin signaling pathways is considered to be among those mechanisms. The PAM pathway has a significant role in controlling cell metabolism, growth, proliferation, and survival. It is one of the main pathways that are responsible for malignant transformation in breast cancer and for its resistance to therapy [6]. The activity of the PAM pathway is enhanced either by the activation of one of the proto-oncogenes (e.g., AKT, PI3K) or by the inactivation of PTEN, a tumor-suppressing component of the pathway [7]. S6 kinase 1 (S6K1) is a key effector of the PAM signaling pathway. Some studies have shown that the activity of S6K1 is increased in cancer cells [8,9].

Wnt signaling pathway is involved in several processes that occur in development of breast cancer, such as proliferation, metastasis, regulation of the immune microenvironment, stemness, therapy resistance, and phenotype shaping [10]. In the canonical Wnt pathway, the signaling cascade results with β-catenin entering the nucleus and interacting with transcription factors, followed by the activation of target genes. Glycogen synthase kinase-3β (GSK3β) is a part of the “destruction complex” that inhibits β-catenin activation and transition to nucleus. The destruction complex is assembled and active as long as the Wnt pathway is not activated by Wnt. Overexpression of β-catenin and its nuclear localization is the common result (hallmark) of the aberrant Wnt pathway activation [11,12]. This may be a consequence of mutations in APC, typical of colorectal carcinoma, AXIN2 in adrenocortical carcinomas or the CTNNB1 gene, coding for β-catenin, in endometrial, hepatobiliary, melanoma, and colorectal cancers [13]. Other than β-catenin, several other components of Wnt signaling pathway (e.g., Wnts and Wnt receptors) tend to be overexpressed in different types of cancer [10,12]. Constituents of the Wnt signaling pathway may also be affected by epigenetic alterations, resulting in upregulation or downregulation [14], whereas the activity of GSK3β is typically regulated by phosphorylation [15]. The activation of this pathway is associated with the development of TNBC phenotype and with a higher metastasis rate and poor prognosis of TNBC [16,17].

PAM and Wnt signaling pathways are interconnected by GSK3β, a key kinase in regulating β-catenin degradation, while its own activity is regulated by kinases from the PAM signaling cascade: S6K1 and AKT [18]. GSK3β also suppresses the activity of PTEN, the PAM pathway suppressor. The interrelation between the PAM and Wnt pathways provided by GSK3β could enable the tumor cells to simultaneously activate both pathways in order to survive. Therefore, GSK3β and S6K1 may be implicated in the progression and development of drug resistance; their simultaneous targeting with combination therapy could potentially be beneficial for TNBC patients.

In this study, we analysed mRNA and protein expression levels of GSK3β and S6K1, as two interacting components of the PAM and Wnt signaling pathways, in TNBC patients. The main goal was to establish whether there is a significant correlation between the expressions of these two genes. Furthermore, we wanted to look at the impact of GSK3β and S6K1 on the severity and prognosis of TNBC and determine whether the expressions of these genes could be used to predict the outcome of the disease.

## 2. Materials and Methods

### 2.1. Patients and Samples

The study includes 119 patients diagnosed with TNBC that were surgically treated at the Institute for Oncology and Radiology of Serbia in Belgrade (IORS) between 2009 and 2014, with a follow-up period being until the end of 2024. The resected tumor samples were formalin-fixed and paraffin-embedded. The diagnoses for each patient were established after the standard examination of hematoxylin and eosin (H&E)-stained tissue microsections, and subsequent IHC analysis of ER, PR, and HER-2 status. The IHC analysis was performed with semi-quantitative commercial IHC assays according to the manufacturer’s instructions and protein expression was assessed by two independent pathologists. The histopathological examination of the tumor and axillary lymph nodes provided the information about the tumor size and grade, nuclear grade, and lymph node status (Table 1).

This study was performed in accordance with the ethical standards of the 1964 Declaration of Helsinki and with the ethical approval from the Ethics Committee of the Institute for Oncology and Radiology of Serbia (approval number: 1669-01). All patients consented to the use of their tissue and associated data in this study.

### 2.2. RNA Extraction

The archived formalin-fixed paraffin-embedded (FFPE) samples of paired tumor and non-tumor breast tissues were used for the extraction of total RNA and further real-time PCR analysis. FFPE tissue was deparaffinized in two subsequent incubations with xylen at 50 °C. It was further rehydrated in subsequent treatments with absolute and 70% diluted ethyl alcohol. Samples were incubated in digestion buffer with proteinase K (500 μg/mL) at 55 °C for 3 h. Further steps of RNA extraction were performed using Trizol (Ambion, Life Technologies, Carlsbad, CA, USA), according to the manufacturer’s instructions. The obtained RNA was dissolved in nuclease-free water (Qiagen, Hilden, Germany) and kept at −80 °C until further analysis. The concentration and quality of the extracted RNA was measured with a nano-drop spectrophotometer with samples varying between 50 and 3000 ng/μL and absorbance ratios within expected values. The integrity of RNA was verified by agarose gel electrophoresis.

### 2.3. Reverse Transcription and Real-Time PCR Analysis

Total RNA (350 ng) was first treated with 1U of RNase-free Dnase I (Thermo Scientific, Vilnius, Lithuania), as described in the manufacturer’s protocol. Reverse transcription was then performed in 20 μL volume with random primers using the High-Capacity cDNA Reverse Transcription Kit (Applied Biosystems, Vilnius, Lithuania). The reaction was carried out in a thermal cycler at 37 °C for 2 h and 85 °C for 5 min. The resulting cDNA was used for a real-time PCR expression analysis of GSK3β (gene GSK3B, Assay ID: Hs01047719_m1) and S6K1 (gene RPS6KB1, Assay ID: Hs00356367_m1) genes on the 7500 Real-Time PCR System (Applied Biosystems) using Taqman technology (Applied Biosystems). Beta actin (Assay ID: Hs01060665_g1) was used as the endogenous control. A real-time PCR analysis was performed in 20 μL volume with 30 ng of cDNA and 1x Taqman Universal PCR Master Mix in each reaction. All samples were run in triplicate, with the temperature profile for the reaction being as follows: 50 °C for 2 min, 95 °C for 10 min, followed by 40 cycles at 95 °C for 15 s and 60 °C for 1 min. Each plate contained non-template controls for each assay. The cycle threshold (Ct) values were calculated in the 7500 Real-Time PCR System SDS v1.4.0 software (Applied Biosystems, Foster City, CA, USA). Analysis of the relative quantification was performed using the 2^−^^ΔΔCt^ method.

### 2.4. Immunohistochemistry

Immunohistochemical analysis was performed on selected FFPE tissue samples of tumor parenchyma, cut to 4 μm sections and mounted on silanized glass slides. The samples were then deparaffinized, rehydrated, and subjected to antigen retrieval in citrate puffer (pH 6), followed by blocking of endogenous peroxidase and nonspecific binding. Sections were then incubated with the primary antibodies, S6K1 (p70 S6 Kinase Polyclonal Antibody, Invitrogen, Rockford, IL, USA) and GSK3β (GSK3 beta antibody, Biorbyt, Cambridge, UK), according to the manufacturer’s instructions. Control tissue sections included third-party liver tissue, according to standard protocols. Visualization was performed through a standard DAB method, while nuclei were contrasted with hematoxylin.

### 2.5. Immunohistochemical Evaluation

Stained tissue was evaluated by two pathologists on at least three representative fields of tumor parenchyma for each sample. Each field was evaluated for the intensity of staining as 0 (negative), 1 (weak), 2 (moderate), and 3 (strong), as well as the percentage of stained cells. Histological score was then calculated as follows: [(% of weak staining × 1) + (% of moderate staining × 2) + (% of strong staining × 3)]. The final H-score was calculated as mean value of scores for all three fields of one sample. The cutoff value for S6K1 was set at 200, as stated in the literature [20]. For GSK3β, the cutoff value was set at 40, i.e., the median H-score of all the samples with positive protein expression, since there is no standardized cutoff value for GSK3β in TNBC.

### 2.6. Statistical Analysis

The results of qPCR were further statistically analysed with the clinical, pathohistological, and immunohistochemistry data of Ki-67, EGFR, PD-L1, and androgen receptor (AR) proteins. Fisher’s exact test was used for association analysis and the Spearman’s rank correlation coefficient was calculated for the assessment of correlation between chosen parameters, since the data did not have a normal distribution. The Wilcoxon signed-rank test was used to calculate the difference between expression levels of GSK3β and S6K1 genes in the paired tumor and normal tissues, since the data were non-parametric and included paired samples of tissue. The log-rank test was used to compare the Kaplan–Meier survival curves in the overall survival (OS) and disease-free interval (DFI) analyses. OS was calculated from the date of the surgery to the date of the last follow-up examination or death of the patient, while DFI was calculated from the date of the surgery to the date of the recorded progression of the disease. The statistical analysis of the obtained data was performed using the GraphPad Prism 8 software for association, correlation, and Kaplan–Meier survival analysis, SigmaPlot 14.0 software (Systat Software Inc., San Jose, CA, USA) was used for the multivariate Cox regression analysis, and R Statistical Software (v4.5.0; R Core Team 2025) was use for more complex Fisher’s exact tests. Statistical differences were considered significant for *p* ≤ 0.05.

## 3. Results

Total RNA was isolated from pairs of archived tumor and normal tissues of patients with TNBC in order to analyze the level of mRNA expression for GSK3β and S6 kinase 1 genes. The analysis of of GSK3β and S6K1 mRNA expression included 119 patients with TNBC. Patients with a minimum two-fold difference in the level of expression of mRNA in tumor tissue compared to normal tissue were considered to have a significant change in the mRNA expression, whether it was an increase or a decrease. Protein expression for S6K1 and GSK3β was performed by immunohistochemical analysis. The cutoff value for S6K1 was H-score of 200, where samples with H-score 200 and higher were considered to have high protein expression, while those with H-score lower than 200 have low expression. For GSK3β, cutoff value was H-score of 40.

### 3.1. mRNA Expression

#### 3.1.1. Expression Analysis and Association Between GSK3β and S6K1

The results of expression analysis performed with qPCR showed that the levels of GSK3β and S6K1 mRNA significantly changed in 43.7% and 25.2% of the patients, respectively. There was a significant decrease in mRNA expression in 41.2% of the patients for GSK3β and 14.3% for S6K1, while a significant increase occurred in 2.5% for GSK3β and 10.9% for S6K1 (Table 2).

**Figure 1:**
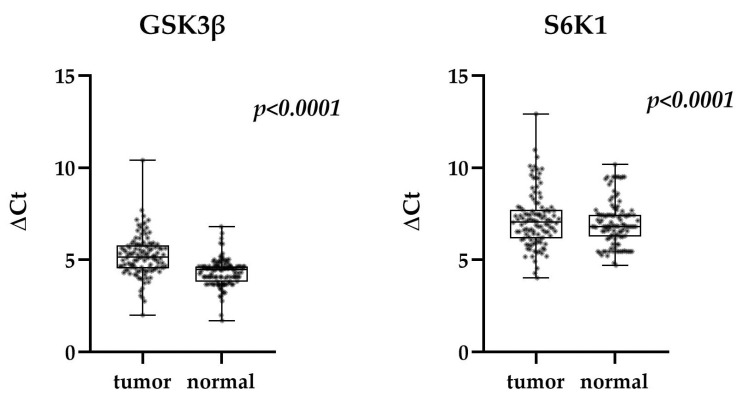
Expression of GSK3β and S6K1 in tumor and normal tissue of TNBC patients (Wilcoxon signed-rank test) in relation to β actin. Median, quartiles maximum, and minimum values are presented.

The Wilcoxon signed-rank test was used to compare expression of GSK3β and S6K1 between paired tumor and normal tissue samples from TNBC patients (Figure 1). The expression was leveled with corresponding expression of β actin and presented as ΔCt value. With median values of 5.146 (tumor) and 4.469 (normal) for GSK3β and 7.057 (tumor) and 6.806 (normal) for S6K1, the expression level of both genes was proved to be significantly lower in tumor tissue compared to normal (*p* < 0.0001 for both GSK3β and S6K1).

The analysis of the association between the levels of expression of GSK3β and S6 kinase 1 by Fisher’s exact test showed a statistically significant association (*p* = 0.0208), indicating the connection between the two pathways. This result was expanded by the Spearman correlation analysis which showed a weak positive correlation (*p* < 0.0001, r = 0.3511) between the mRNA expression of GSK3β and S6K1 (Table 3). The existing correlation unequivocally confirmed the link between PAM and Wnt pathways.

#### 3.1.2. Association Between GSK3β and S6K1 and Other Parameters

In further analyses, Fisher’s exact test was performed to assess the association of GSK3β and S6K1 mRNA levels with the relevant clinicopathologic parameters such as tumor size, tumor grade, nuclear grade, extirpated lymph node status, presence of locoregional recurrencies, distant metastases, and application of adjuvant radiotherapy. We also looked at the association of gene expression with protein expression levels of PD-L1, EGFR, androgen receptor (AR), and Ki-67. The expression analysis of these proteins was previously performed by immunohistochemistry [19]. The results showed that the change in expression, both increase and decrease, of S6K1 was associated with lower levels of AR (*p* = 0.0238). Also, a decreased level of S6K1 showed an association with increased EGFR expression (*p* = 0.0027). Moreover, the Spearman correlation analysis showed there was a statistically significant level of negative correlation between S6K1 and EGFR (*p* = 0.004, r = −0.2618). Expression within normal range of GSK3β showed association with low EGFR (*p* = 0.0454) and low PD-L1 (*p* = 0.0373). All the results of the association analysis are shown in Table 4.

The existence of a positive correlation between GSK3β and S6K1 and a significant association of S6K1 with AR and EGFR, raised the question of whether there was any association of simultaneous expression of GSK3β and S6K1 with the two receptor proteins. The analysis of simultaneous changes in expression of GSK3β and S6K1 also showed the association with the expression of AR and EGFR. Specifically, the change in mRNA level of both GSK3β and S6K1 was associated with low expression of AR (*p* = 0.0057), while the simultaneous decrease in both mRNA levels showed association with high EGFR (*p* = 0.0153), which was consistent with the relation of S6K1 to AR and EGFR. We did not find association of the expression of GSK3β and S6K1 with any other clinicopathologic parameter (Table 5).

We further assessed association between both GSK3β and S6K1 expression and previously defined “high risk” profile of TNBC, which included low expression of PD-L1, low expression of AR and high expression of EGFR [19]. Fisher’s exact test showed a significant association of the “high risk” phenotype with low expression of S6K1, but also with the simultaneous decrease in expression of both GSK3β and S6K1 (see Table 6). This result added to the complexity of the “high risk” genetic profile of TNBC.

### 3.2. Protein Expression

**Figure 2:**
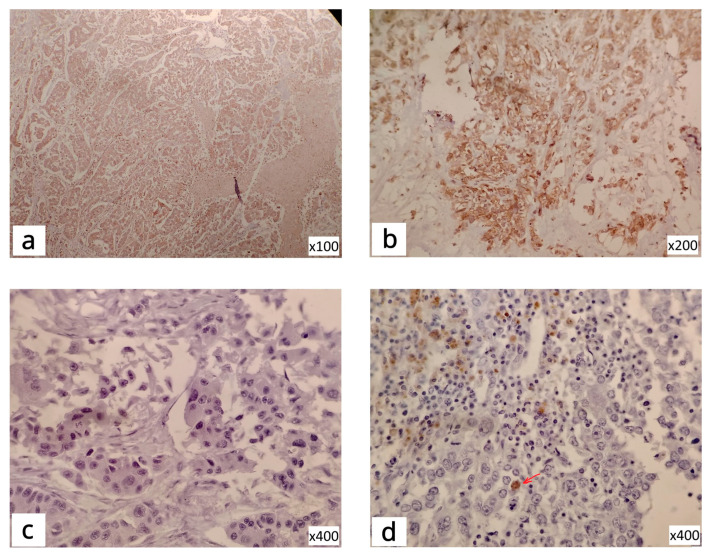
IHC staining of TNBC tissue: S6K1-cytoplasmic reactivity (**a**); S6K1-positive cytoplasmic and nuclear reactivity (**b**); GSK3β-negative reactivity (**c**); GSK3β-positive focal nuclear reactivity (**d**), The arrow is to see the difference from negative (**c**).

The results of the IHC protein expression analysis (Figure 2) showed that levels of GSK3β and S6K1 varied significantly among patients. Namely, 82.2% of TNBC showed low level of GSK3β while 17.8% had high protein expression. At the same time, 49.2% of these patients showed low S6K1 protein expression and 50.8% had high IHC expression. The main purpose of this analysis was to determine whether IHC protein expression follows the pattern of mRNA expression within our separate groups, lower and higher expression.

The analysis of protein expression of S6K1 showed that there was a significant association of mRNA expression with the level of protein expression within the group with lower (*p* = 0.0184) and the group with higher mRNA expression (*p* = 0.0373).

The IHC analysis of GSK3β clearly highlighted the overall low protein expression in this cohort. Moreover, 81.6% of patients with low GSK3β mRNA expression, also had low protein expression, showing a statistically significant association between levels of expression of mRNA and protein (*p* = 0.0026).

### 3.3. Survival Analysis

Survival analysis was performed with log-rank test, using Kaplan–Meier survival curves. The comparison was made between different levels of mRNA expression for GSK3β and S6K1 individually as well as for the simultaneous changes in expression of both genes. Overall survival and disease-free interval were measured from the day of surgery to the date of either death for OS or recurrence of the disease for DFI. In cases without events, date of the last checkup was used for calculation.

When patients with low expression of both GSK3β and S6K1 were compared to all other patients, there was a statistically significant difference between disease-free interval of the two groups (*p* = 0.0480), where recurrence occurred in 50% (6/12) of patients with low expression of both genes, and only 22.4% (24/107) of all other patients. This result underlined the significance of the low GSK3β/low S6K1 phenotype for the bad outcome of TNBC.

Similar comparison including low GSK3β/low S6K1 patients with the group of patients with unaltered level of expression of both genes, showed a marginal significance also for disease-free interval (*p* = 0.0516), with recurrence rate of 21.2% (11/52) in the group of patients with no change in expression.

**Figure 3:**
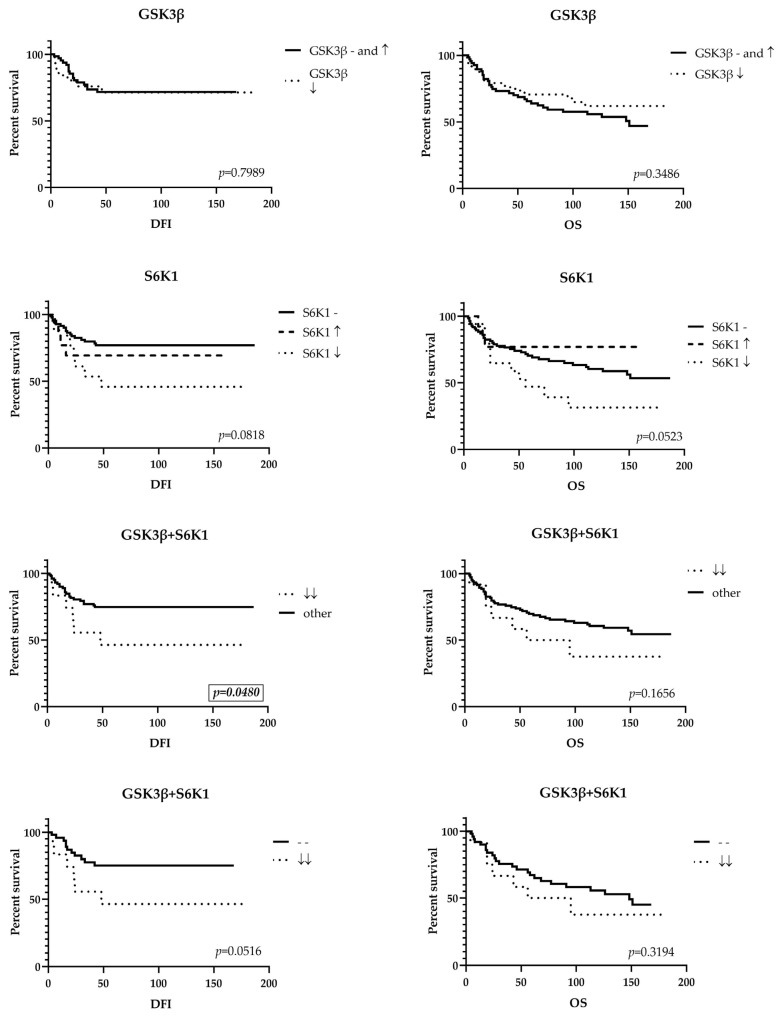
Kaplan–Meier survival curves. DFI, disease-free interval; OS, overall survival; (-) no change in expression; (↑) high expression; (↓) low expression; (↓↓) low expression of both genes.

The overall survival for S6K1 showed a marginally significant result (*p* = 0.0523) with fatal outcome being most frequent in the group of patients with lower level of S6K1 expression, 64.7%, while 39.3% patients without change in expression and 23.1% of patients with higher level of expression had died in the same period of time (Figure 3).

To assess the independent prognostic effect of studied genes and histopathological and clinical parameters, we performed a multivariate Cox regression analysis. It showed that increased tumor size as well as positive lymph node status are independent prognostic factors that reduce the disease-free interval (HR = 10.207, *p* = 0.003, 95% CI 2.262–46.060 and HR = 3.088, *p* = 0.009, 95% CI 1.323–7.207, respectively) and overall survival (HR = 7.326, *p* < 0.001, 95% CI 2.504–21.430 and HR = 2.196, *p* = 0.020, 95% CI 1.130–4.267, respectively), while the application of adjuvant radiotherapy independently positively affects the duration of DFI (HR = 0.409, *p* = 0.028, 95% CI 0.184–0.909).

## 4. Discussion

Triple-negative breast cancer makes up a less common (15–20%) but particularly difficult type of breast cancer, since it is recognized as the most aggressive type, with high frequency of recurrence, metastasis, and mortality. The absence of estrogen, progesterone, and HER2 receptors at the surface of cancer cells prevents TNBC patients from being treated with targeted therapy, with the exception of a few target-specific drugs (eg. PARP and immune checkpoint inhibitors) which are applicable for only a part of the TNBC-affected population. The lack of a specific therapy, along with the common occurrence of developing resistance to conventional therapies, presents a big challenge in the treatment of TNBC. A large number of clinical trials evaluating different molecular targets (AR, PAM pathway, Notch pathway, etc.) have already been conducted or are in progress but have not produced significant results [4,21]. The main reason for this is extreme heterogeneity of TNBC, with a number of molecular subtypes that need precise molecular characterization in order for adequate therapeutic protocols to be defined in the future. The research that has been performed in deciphering the phenomenon of resistance has shown that the malignant breast cancer cells can use several different mechanisms en route to obtaining the resistant phenotype [5]. That leads to the conclusion that overcoming therapy resistance should include targeting more than one mechanism of acquiring resistance, so that compensatory pathways would not lead to further unresponsiveness to therapy. Research has shown that single-agent efficacy is limited, and the current approach to creating therapeutic protocols is to design combinations of therapeutical options [21].

The PAM and Wnt pathways are reported to be responsible for the development of resistance to therapy among TNBC patients [5,22]. In addition, their simultaneous abrogation restores sensitivity to therapy [23]. With that in mind, our study investigated the question of whether the existence of synergistic contribution of the two pathways to the malignant phenotype could be evaluated at the level of mRNA expression of GSK3β and S6K1, where the two pathways converge.

Our findings undeniably show that the levels of mRNA expression of GSK3β and S6K1 are positively correlated, which implies that there is a coordinated regulation of gene expression between the two pathways, confirming the connection between them.

We also established that patients with simultaneous downregulation of mRNA expression of both GSK3β and S6K1 genes have worse outcomes than patients with an unchanged or higher level of expression. The expression of GSK3β and S6K1 did not show significant association with any of the clinicopathologic parameters. However, we found an association with the expression of AR and EGFR; moreover, we found an association with a specific expression profile of PD-L1, EGFR, and AR proteins that we defined as a “high-risk phenotype” in our previous research [19]. This indicates that GSK3β and S6K1 may also contribute to the “high-risk phenotype” and that an expanded version would comprise the following attributes: low expression of PD-L1, low expression of AR, and high expression of EGFR proteins, along with low expression of both GSK3β and S6K1 mRNA.

The dual nature of GSK3β, its capability of being both an oncogene and a tumor suppressor [15,24,25], enables it to behave differently in different types of cancer. That implies that the level of expression of GSK3β could vary depending on its role in a specific cell. There is evidence that GSK3β can act as an oncogene as well as a tumor suppressor in breast cancer, although it is predominantly considered to be a tumor suppressor [26,27]. This is consistent with our results, where GSK3β was overexpressed in only 2.5% of patients, while it had decreased mRNA expression in 41.2% of TNBC patients. The low expression of GSK3β was shown to be unfavorable in our cohort, which would suggest it has a tumor suppressor role in TNBC. To determine whether the results obtained on mRNA level can apply for functional proteins, we introduced an IHC protein expression analysis, which showed the association of the low expression of proteins with the low expression of mRNA, confirming the overall low expression of GSK3β.

On the other hand, multiple studies have shown that the protein expression of S6K1 is upregulated in breast cancer [8,9,28,29], although there are some studies that report a positive impact of the expression of phosphorylated S6K1 on survival [30,31], which was specifically shown in breast cancer, in contrast to other types of cancer [32]. Given that S6K1 is capable of indirectly inhibiting Akt through a negative feedback mechanism [33], the downregulation of S6K1 could cause the activation of the Akt pathway, generating an oncogenic effect. There is evidence that GSK3β can either activate or inhibit the PAM pathway, resulting in the phosphorylation or dephosphorylation of S6K1, which solely depends on the specific context of a given cell [34]. Our results are consistent with these findings, considering that we detected downregulated mRNA expression of S6K1 in 14.3% of patients and upregulated mRNA expression of S6K1 in 10.9% of patients. These results are in correlation with the IHC results for protein expression. Namely, our analysis of the expression of S6K1 protein showed that there is association between mRNA and protein expression in both groups, with mRNA expression deviating from normal expression. This indicates that the low expression of the S6K1 protein could also be viewed as an indicator of a bad outcome. Therefore, results from our cohort support the opinion that the downregulation of S6K1 could have an oncogenic effect and is unfavorable for TNBC patients.

We must add that there are different levels of protein expression of S6K1 in the group of patients with unchanged levels of mRNA expression; this is not surprising given the high criterion for the cutoff value of mRNA expression, since we considered no less than a two-fold change to be significant. Moreover, approximately an equal number of these patients have either high or low protein expression, so we can argue whether this method (mRNA expression) could be used as a definitive tool in diagnostics. However, the question remains of whether the gene mRNA expression level is directly proportional to the protein level in the cell as a rule. There are mechanisms of posttranslational regulation that influence the level of translational efficiency, determining the productivity of mRNA [35,36,37].

mRNA expression did not show an association with any of the clinicopathological parameters. However, it was associated with markers of bad outcome (EGFR, AR and PD-L1). Therefore, the survival analysis performed in this study confirmed the malign effect of the low expression of both GSK3β and S6K1. Individually, variations in the expressions of GSK3β and S6K1 did not cause significant changes in either overall survival or recurrence, but we found that recurrence of the disease was more frequent in patients with a simultaneous drop in mRNA expression of GSK3β and S6K1. This result should be considered to support the existance of the synergistic effect of the PAM and Wnt pathways on the outcome of TNBC.

In conclusion, our study stipulates that a positive correlation exists between mRNA expression of GSK3β and S6K1, showing that there is probably a coordinated regulation of transcription of these genes. The low mRNA levels of both GSK3β and S6K1 are shown to have adverse significance in context of TNBC. Moreover, the protein level follows the pattern of mRNA expression in both genes, implying that both proteins act as tumor suppressors in this context. The correlation shown between expression patterns of GSK3β and S6K1 on one side and EGFR, AR, and PD-L1 on the other suggests that, to some extent, we could possibly treat GSK3β and S6K1 as accompanying markers for the identification of TNBC patients with a particularly high-risk condition.

## Figures and Tables

**Table 1:** Patients’ characteristics.

Features	Number of Patients
Tumor size (cm)	
≤2	36 (30.3%)
2 ≤ 5	72 (60.5%)
>5	11 (9.2%)
Tumor grade	
G1	0
G2	55 (46.2%)
G3	64 (53.8%)
Nuclear grade	
NG1	4 (3.4%)
NG2	50 (42.0%)
NG3	65 (54.6%)
Lymph node status	
Positive	42 (35.3%)
Negative	76 (63.9%)
NA	1 (0.8%)
Relapse	
Yes	30 (25.2%)
No	89 (74.8%)
Locoregional recurrence	
Yes	13 (10.9%)
No	106 (89.1%)
Distant metastases	
Yes	26 (21.8%)
No	93 (78.2%)
Adjuvant radiotherapy	
No	42 (35.3%)
Yes	74 (62.2%)
NA	3 (2.5%)
PD-L1 *	
High	49 (41.2%)
Low	70 (58.8%)
AR *	
High	54 (45.4%)
Low	65 (54.6%)
EGFR *	
High	38 (31.9%)
Low	81 (68.1%)
Ki67 *	
Low	15 (12.6%)
Intermediate	9 (7.6%)
High	95 (79.8%)

Note: *—results of IHC analysis, previously published in [19].

**Table 2:** Results of the expression analysis.

Gene	Number of Patients
GSK3β	
No change	67 (56.3%)
High	3 (2.5%)
Low	49 (41.2%)
S6K1	
No change	89 (74.8%)
High	13 (10.9%)
Low	17 (14.3%)

**Table 3:** Association and correlation between expression of GSK3β and S6K1.

		GSK3β		
		Normal	High	Low
**S6K1**	Normal	52	2	35
	High	10	1	2
	Low	5	0	12
		***p*** **=** **0.0208**		
Spearman correlation				
***p*** **<** **0.0001**		r = 0.3511		

Clarification: Normal—same level of expression as in normal breast tissue of the same patient. Bold indicates statistically significant values *p* ≤ 0.05.

**Table 4:** Association between mRNA expression of GSK3 and S6K1 and clinicopathological and immunohistochemical characteristics of TNBC.

Tumor Features	GSK3β				S6K1			
	Normal	High	Low	*p* Value	Normal	High	Low	*p* Value
Tumor size				0.5291				0.9047
≤2 cm	22 (18.5%)	0 (0%)	14 (11.8%)		29 (24.4%)	3 (2.5%)	4 (3.4%)	
2 ≤ 5 cm	38 (31.9%)	2 (1.7%)	32 (26.9%)		52 (43.7%)	9 (7.6%)	11 (9.2%)	
>5 cm	7 (5.9%)	1 (0.8%)	3 (2.5%)		8 (6.7%)	1 (0.8%)	2 (1.7%)	
Tumor grade				>0.9999				0.358
G1	0 (0%)	0 (0%)	0 (0%)		0	0	0	
G2	31 (26.1%)	1 (0.8%)	23 (19.3%)		41 (34.5%)	8 (6.7%)	6 (5.0%)	
G3	36 (30.3%)	2 (1.7%)	26 (21.8%)		48 (40.3%)	5 (4.2%)	11 (9.2%)	
Nuclear grade				0.6195				0.132
NG1	3 (2.5%)	0 (0%)	1 (0.8%)		3 (2.5%)	0 (0%)	1 (0.8%)	
NG2	30 (25.2%)	1 (0.8%)	19 (16.0%)		37 (31.1%)	9 (7.6%)	4 (3.4%)	
NG3	34 (28.6%)	2 (1.7%)	29 (24.4%)		49 (41.2%)	4 (3.4%)	12 (10.1%)	
LNS				0.1219				0.5352
Positive	38 (31.9%)	3 (2.5%)	35 (29.4%)		59 (49.6%)	7 (5.9%)	10 (8.4%)	
Negative	29 (24.4%)	0 (0%)	13 (10.9%)		29 (24.4%)	6 (5.0%)	7 (5.9%)	
NA	0	0	1 (0.8%)		1 (0.8%)	0	0	
Relapse				>0.9999				0.1523
Yes	16 (13.4%)	1 (0.8%)	12 (10.1%)		18 (15.13%)	4 (3.36%)	7 (5.88%)	
No	51 (42.9%)	2 (1.7%)	37 (31.1%)		71 (59.66%)	9 (7.56%)	10 (8.40%)	
LR				0.7695				0.6743
Yes	7 (5.88%)	0 (0%)	6 (5.04%)		8 (6.7%)	2 (1.7%)	2 (1.7%)	
No	60 (50.42%)	3 (2.521%)	43 (36.13%)		81 (68.1%)	11 (9.2%)	15 (12.6%)	
DM				0.5043				0.3976
Yes	16 (13.4%)	1 (0.8%)	9 (7.6%)		17 (14.3%)	4 (3.4%)	5 (4.2%)	
No	51 (42.9%)	2 (1.7%)	40 (33.6%)		72 (60.5%)	9 (7.6%)	12 (10.1%)	
AdjR				>0.9999				0.3216
No	25 (21.0%)	0 (0%)	17 (14.3%)		35 (29.4%)	2 (1.7%)	5 (4.2%)	
Yes	41 (34.5%)	3 (2.5%)	30 (25.2%)		52 (43.7%)	10 (8.4%)	12 (10.1%)	
NA	1 (0.8%)	0	2 (1.7%)		2 (1.7%)	1 (0.8%)	0	
PD-L1				**0.0373**				0.2472
High	22 (18.5%)	1 (0.8%)	26 (21.8%)		40 (33.6%)	5 (4.2%)	4 (3.4%)	
Low	45 (37.8%)	2 (1.7%)	23 (19.3%)		49 (41.2%)	8 (6.7%)	13 (10.9%)	
AR				0.0556				**0.0238**
High	30 (25.2%)	1 (0.8%)	13 (10.9%)		39 (32.8%)	2 (1.7%)	3 (2.5%)	
Low	37 (31.1%)	2 (1.7%)	36 (30.3%)		50 (42.0%)	11 (9.2%)	14 (11.8%)	
EGFR				**0.0454**				**0.0027**
High	17 (14.3%)	0 (0%)	21 (17.6%)		26 (21.8%)	1 (0.8%)	11 (9.2%)	
Low	50 (42.0%)	3 (2.5%)	28 (23.5%)		63 (52.9%)	12 (10.1%)	6 (5.0%)	
Ki67				0.1573				0.1665
Low	12 (10.1%)	0 (0%)	3 (2.5%)		14 (11.8%)	1 (0.8%)	0 (0%)	
Intermediate	3 (2.5%)	1 (0.8%)	5 (4.2%)		6 (5.0%)	0 (0%)	3 (2.5%)	
High	52 (43.7%)	2 (1.7%)	41 (34.5%)		69 (58.0%)	12 (10.1%)	14 (11.8%)	

Abbreviations: LNS, lymph node status; LR, locoregional recurrence; DM, distant metastases; AdjR, adjuvant radiotherapy. In statistical analysis of data for GSK3β, we considered both normal and high expression as a single group, with expression within normal range. Bold indicates statistically significant values *p* ≤ 0.05.

**Table 5:** Association of simultaneous expression of GSK3β and S6K1 with the characteristics of TNBC.

	GSK3β/S6K1		
	-/-	↓/↓	*p* Value
**Tumor size**			>0.9999
≤5 cm	47	11	
>5 cm	5	1	
**Tumor grade**			>0.9999
G1 + G2	25	6	
G3	27	6	
**Nuclear grade**			0.7517
NG1 + NG2	26	5	
NG3	26	7	
**Lymph node status**			>0.9999
Positive	21	5	
Negative	31	7	
**Relapse**			0.1559
Yes	11	5	
No	41	7	
**Locoregional recurrence**			0.6067
Yes	5	2	
No	47	10	
**Distant metastases**			0.7154
Yes	11	3	
No	41	9	
**Adjuvant radiotherapy**			0.2017
Yes	22	8	
No	29	4	
**PD-L1**			>0.9999
High	18	4	
Low	34	8	
**AR**			**0.0083**
High	27	1	
Low	25	11	
**EGFR**			**0.0162**
High	14	8	
Low	38	4	
**Ki67**			>0.9999
High	15	3	
Low	37	9	

Note: (-) same level of expression as in normal breast tissue of the same patient; (↓) low expression. Bold indicates statistically significant values *p* ≤ 0.05.

**Table 6:** Association between expression of GSK3β and S6K1 and simultaneous expression of PD/L1, AR, and EGFR proteins.

		PD-L1/AR/EGFR		
		High-Risk	Other	*p* Value
		Phenotype	Phenotypes	
**GSK3β**	-	7	60	0.4675
	↑	0	3	
	↓	9	40	
**S6K1**	-	10	79	**0.0137**
	↑	0	13	
	↓	6	11	
**GSK3β/**	-/-	6	46	**0.0252**
**S6K1**	↓/↓	5	7	

Note: (-) same level of expression as in normal tissue of the same patient; (↓) lower expression; (↑) higher expression. *High-risk phenotype:* low expression of PD-L1 and AR and high expression of EGFR. *Other phenotypes:* all other combinations of PD-L1, AR, and EGFR expressions. Bold indicates statistically significant values *p* ≤ 0.05.

## Data Availability

The data presented in this study are available on request from the corresponding author.

## Abbreviations

APC	Adenomatous polyposis coli
AR	Androgen receptor
AXIN2	Axis inhibition protein 2
cDNA	Complementary DNA
CI	Confidence interval
DAB	3,3′-diaminobenzidine
DFI	Disease-free interval
EGFR	Epidermal growth factor receptor
ER	Estrogen receptor
FFPE	Formalin-fixed paraffin-embedded
GSK3 beta	Glycogen synthase kinase-3 beta
H&E	Hematoxylin and eosin
HER-2	Human epidermal growth factor receptor 2
HR	Hazard ratio
IHC	Immunohistochemistry
IORS	Institute for Oncology and Radiology of Serbia
mRNA	Messenger ribonucleic acid
mTOR	Mammalian target of rapamycin
OS	Overall survival
PAM	PI3K-Akt-mTOR
PCR	Polymerase chain reaction
PD-L1	Programmed death-ligand 1
PI3K	Phosphoinositide 3-kinase
PR	Progesterone receptor
PTEN	Phosphatase and tensin homolog
S6K1	S6 kinase 1
TNBC	Triple-negative breast cancer
